# Retroversion of the Impregnated Uterus and Spontaneous Reposition

**Published:** 1868-10

**Authors:** C. C. F. Gay


ART. II.—*Retroversion of the Impregnated Uterus and Spontaneous Reposition.* BY C. C. F. GAY, M. D.

I transcribe from my note-book the following interesting and instructive case of retroversio-uteri, although a faithful record of it is somewhat disparaging to the method employed and pursued in the management of the case. I record the case, however, most willingly, believing that he who accurately and honestly details a case under his observation and treatment, contributes thereby something to the general stock of medical knowledge, which entitles him to a hearing:

I was consulted on April 28th, 1862, by Mrs. F., aged 31 years. She states that her health has been impaired for a long time, that leucorrhœa has troubled her for three years; has one child, now six years old; never since been pregnant; never had miscarriage; thinks she has scrofula; three of her relatives had been troubled with uterine disease, of which one died. Present symptoms—leucorrhœa; frequent desire to urinate; pain in right side and back; pulse frequent; tongue coated; no appetite; bowels regular; difficulty in walking; general malaise. Examination with speculum showed extensive ulcerations of the os, a portion of which presented the strawberry appearance, and bleeds to the touch. Applied argt. nitras.

May 5th. Considerable hemorrhage followed the application made on April 28th. Introduced Simpson's sound and found uterus retroverted. Reposited the uterus with the sound, and directed patient to lie upon her back for a few hours.

May 8th. Uterus again displaced; again brought up fundus to its normal position with the sound.

May 11th. Patient has remained in bed and had considerable pain; some nausea and vomiting; womb still retroverted. Again used the sound, but shall not attempt again to reposit the womb until the ulcerations are healed. Applied nit. silver; afterwards cotton pessary, saturated with sweet oil, and prescribed iod. pot. grs. iij, ter die, and cold water vaginal injections.

May 23d. Considerable bloody discharge after local application; shall make the application in the future lighter and more cautiously.

May 27th. The os looking better; bloody discharge continued for two days after last application; used to-day cotton ball saturated with ol. olive and tr. iodine, equal parts, and morph. gr. ss.

June 7th. On examination to-day discover that the womb has been spontaneously reposited. The os so far inclined backwards towards the sacrum as to occasion some difficulty in finding it; normal position of the womb ascertained by the use of the sound. Patient much improved; pain abated; no difficulty in urinating; ulcerations nearly healed; not nearly so much leucorrhœa. Continue the cotton ball, and ordered its removal in twenty-four hours, unless its presence becomes uncomfortable before the expiration of this time. After its removal, cold water injections. Discontinue medicine.

June 23. Uterus remains in normal position and patient much improved.

July 3d. Continue treatment. Applied carefully argt. nitras. Has now no discharge, either leucorrhœal or blood.

July 21st. Argt. nit. has been applied once each week; patient improving; scarcely any disease remaining of the os.

Decided a month ago, nearly, or on June 7th, at the time I found the uterus in its normal position, that this woman was pregnant, and again to-day reiterated the same opinion, and am positive as to the correctness of the diagnosis, although patient will not believe it. There has been no menstruation, although she thinks she has menstruated regularly.

August 23d. Patient has come to the conclusion that I am correct in my views as to her pregnancy, as she believes she feels motion. Made no more applications to the os. Patient goes out and feels well.

Nov. 3d. I delivered this female at full term of a living male child, weighing ten pounds. Both mother and child doing well.

The failure to diagnose early the pregnancy of this woman is not the most interesting feature in making up the record of this case. Had the incomplete diagnosis led to miscarriage I doubtless should have excused myself from reporting it, but as the error did not lead to any such result, I am quite willing to report the error, and to believe that a more interesting feature than that of failure to diagnose presents itself in the fact that a gravid uterus was able to sustain such manipulations with impunity. There were good and sufficient reasons why pregnancy was not diagnosticated. Six years had intervened since the last pregnancy; there was retroversion of the uterus and extensive ulcerations of the os and cervix, with regular menstruation. And a firm denial, on the part of the patient herself, that there was a possibility or probability of pregnancy. These several circumstances led me into the error of treating the case precisely as though pregnancy did not exist in reality.

It has been asserted that considerable ulceration of the os with much leucorrhœa precluded the possibility of pregnancy. It has been asserted, moreover, that a retroverted uterus could not become impregnated; facts presented in this single case fully controverts the theory that diseased os and misplaced womb must of necessity always subject the female to the sterile condition. I have sufficient evidence to warrant the assertion that this lady's womb was retroverted before the pregnancy, and that it had been so displaced since her last confinement; for notwithstanding this patient has since been in labor with a still-born child at term, the uterus at this present writing is still retroverted, and became so after the birth of both of the last two children, although every precaution to prevent and guard against such displacement was made use of. On examination I have found the uterus retroverted as at first, and there in its chosen dislocated position it remains, and refuses to respond to any means, mechanical or otherwise, for its permanent reposition, except it be pregnancy.

The retroverted impregnated uterus is capable, as shown in this case, of accomplishing what the unimpregnated uterus is incapable of doing, viz: of spontaneously repositing itself. This is an interesting fact, and may ultimately lead the way to successful treatment for the permanent relief of this form of displacement, for if the uterus may be made to occupy its normal position, and to remain in its proper relations with the other viscera within the pelvis during utero-gestation, why may not the unimpregnated uterus be made to do the same by means of appropriate mechanical appliances, or by appeal to the resources of surgery?

The case which I have recorded opens up the mooted question of the propriety of cauterizing the os in the case of gravid uterus and the probabilities of risk to the fœtus in utero and induction of miscarriage by the use of local applications. When, during pregnancy, there is inconsiderable disease of the os, or cervix, or both, are we justifiable in making local applications? I answer, no; but when there exists considerable disease we are undoubtedly authorized to treat the case only in a more mild and cautious manner, just the same as though we were treating the os or cervix of a non-impregnated uterus.

This recorded case is instructive. I was taught the lesson never to introduce the sound within the cavity of the womb in any case in which there could be the least shadow of doubt cast upon the question of pregnancy. In this instance Simpson's sound was used and introduced with facility within the uterine cavity, upon which the reposition was accomplished. In the future I should incur no risk, for the reason that I have abandoned the use of this sound in place of which Sims' silver probe is now substituted, but which I never employ, as a rule, for this purpose.

Here, then, was a woman pregnant, conception having taken place about the beginning of the month of February. On May 5th, 8th and 11th, the displacement was temporarily rectified by the use of Simpson's sound, at the same time nitrate of silver in substance was applied thoroughly to the diseased os, and both these manipulations were done with impunity; still this experience cannot be distorted into justification for rough handling of a gravid uterus. That miscarriage was not the result of such manipulation was certainly no fault of mine, and my single consolation is in the fact that I am not the only person, who, has at least once in his professional career, committed the mistake of making hasty and incomplete diagnosis.

---

Chloride of copper is now extensively used in Germany against the cattle plague, or rather as a preservative.

